# mRNA-engineered T lymphocytes secreting bispecific T cell engagers with therapeutic potential in solid tumors

**DOI:** 10.3389/fimmu.2025.1684655

**Published:** 2025-11-18

**Authors:** Ivana Zagorac, Ángel Ramírez-Fernández, Antonio Tapia-Galisteo, Laura Rubio-Pérez, Marina Gómez-Rosel, Montserrat Grau, José Luis Rodríguez-Peralto, Luis Álvarez-Vallina, Belén Blanco

**Affiliations:** 1 Cancer Immunotherapy Unit, Department of Immunology, Hospital Universitario12 de Octubre, Madrid, Spain; 2 Immuno-Oncology and Immunotherapy Group, Instituto de Investigación Sanitaria 12 de Octubre (imas12), Madrid, Spain; 3 Centro Nacional de Investigaciones Oncológicas-Hospital del Mar Research Institute Barcelona (CNIO-HMRIB) Cancer Immunotherapy Clinical Research Unit, Centro Nacional de Investigaciones Oncológicas (CNIO), Madrid, Spain; 4 Animal Facility, Instituto de Investigación Sanitaria 12 de Octubre (imas12), Madrid, Spain; 5 Department of Pathology, Hospital Universitario 12 de Octubre, Madrid, Spain; 6 Department of Pathology, Universidad Complutense, Madrid, Spain; 7 Cutaneous Oncology Group, Instituto de Investigación Sanitaria 12 de Octubre (imas12), Madrid, Spain; 8 Centro de Investigación Biomédica en Red en Oncología (CIBERONC), Madrid, Spain; 9 Banc de Sang i Teixits, Barcelona, Spain; 10 Red Española de Terapias Avanzadas (TERAV), Instituto de Salud Carlos III, Madrid, Spain

**Keywords:** mRNA, T cell engager (TCE), CAR-T cell, STAb-T cell, STAR-T cell, solid tumors

## Abstract

**Background:**

In the last decade, c*himeric antigen receptor* (CAR)-modified T cells have revolutionized the treatment of hematologic malignancies. However, antitumor responses in solid tumors remain poor, and the difficulty in finding truly tumor-specific target antigens leads to a high risk of on-target/off-tumor toxicity. Transient modification with mRNA is gaining momentum as an alternative approach to viral transduction in order to achieve a better safety profile. On the other hand, generation of T cells secreting bispecific T cell engagers (TCEs) has been reported to outperform the antitumor efficacy of T lymphocytes expressing membrane-anchored CARs, due to the ability of the soluble TCEs to recruit unmodified bystander T cells.

**Methods:**

We have electroporated human primary T cells with *in vitro* transcribed mRNA encoding an anti-EGFR x anti-CD3 bispecific T cell engager. Such mRNA-modified T cells (STAR^EGFR^-T cells) have been analyzed for anti-EGFR bispecific TCE secretion and for their ability to drive anti-tumor responses against EGFR-expressing cells, both *in vitro* and *in vivo*.

**Results:**

STAR^EGFR^-T cells transiently secrete bispecific TCEs capable of redirecting T lymphocytes to exert tumor cell-specific killing in *in vitro* assays. Moreover, STAR^EGFR^-T cells efficiently control tumor growth in *in vivo* xenograft models of solid malignancy.

**Conclusions:**

Our results strongly support mRNA-engineered TCE-secreting T cells as a promising therapeutic strategy for solid tumors.

## Introduction

Adoptive cell immunotherapy with engineered T cells has revolutionized the landscape of cancer therapy. Treatment of hematological malignancies with chimeric antigen receptor (CAR) T-cells has provided impressive results, and T lymphocytes modified to Secrete bispecific T cell engager (TCE) Antbodies, termed STAb-T cells, have demonstrated superior efficacy to CAR-T cells (1–4) or to complement CAR antitumor activity (5, 6) in animal models, and are now being tested in clinical trials (7). However, most CAR-T therapies against solid tumors have demonstrated only modest therapeutic activity, and the risk of uncontrollable off-tumor toxicities, due to the difficulty in defining truly tumor-specific antigens, hinders its application in the treatment of non-hematological cancers. In this regard, the stable integration of the transgene and persistent modification achieved with the use of viral vectors can lead to exacerbated toxicities against healthy tissues expressing the target antigen. Therefore, transient CAR or TCE expression from mRNA may provide a better safety profile, as potential adverse events, such as on-target/off-tumor toxicities or cytokine release syndrome, would be controlled by discontinuing the infusions (8). In addition, mRNA modification would eliminate other safety concerns associated with retro/lentiviral modifications such as genomic integration and, therefore, potential malignant transformation. In this regard, the Food and Drug Administration has recently expressed concern about the development of T-cell malignancies following BCMA and CD19 targeting CAR-T therapies (9). On the other hand, clinical-grade mRNA manufacturing process is faster, and more cost-effective and straightforward than the production of integrating viral vectors. Finally, the use of mRNA would reduce the tight regulation associated with genetically engineered cell therapies, which delays the clinical translation of novel CAR-T cell approaches (10).

The development of mRNA therapeutics has been prompted by the success of SARS-COV2 vaccines, and mRNA-based T cell modification is emerging as an alternative to overcome the challenges associated with viral-based T cell immunotherapies, making this strategy a good approach to test the safety of T cell products in first-in-human clinical trials. A number of preclinical studies have demonstrated the efficacy of mRNA-modified transiently-redirected T cells in different hematologic and solid tumor models (2, 11–14). However, limited positive responses to mRNA CAR-T therapy have been shown in clinical trials. To overcome this limited efficacy, the emerging STAb-T strategy (1, 4, 15, 16), may compensate for the transient nature of mRNA modification with enhanced anti-tumor activity. Thus, we and others have demonstrated that lentivirally-engineered STAb-T cells outperform CAR-T therapy in several *in vivo* models of hematological tumors (1, 2, 4). This superiority derives, at least in part, from the ability of the secreted TCEs to recruit unmodified bystander T cells. Similarly, mRNA-engineered STAb-T cells, termed STAb-mRNA or STAR-T cells, may overcome the limited efficacy of mRNA CAR-T cells by providing enhanced anti-tumor activity despite transient expression. mRNA-modified T cells secreting anti-CD19 × anti-CD3 TCEs have shown potent anti-leukemic effects (2, 14), with one study reporting complete remission and superior efficacy compared with mRNA CAR-T cells (2). In addition, γδ T cells engineered with mRNA to simultaneously express an HLA‐G‐CAR and a PD‐L1/CD3*ϵ* TCE inhibited solid tumor growth (17).

Here we report, for the first time, the generation of mRNA engineered STAR-T cells targeting a solid tumor without concomitant CAR expression, with their efficacy relying solely on the activity of the secreted TCE. Specifically, we have electroporated T cells with an *in vitro* transcribed (IVT) mRNA encoding an anti-EGFR x anti-CD3 in light T cell engager (LiTE) format (18). Such STAR^EGFR^-T cells demonstrated their efficacy *in vitro* and *in vivo*, providing proof of concept that STAR-T cells efficiently produce functional TCEs and, despite transient secretion, may be an effective treatment platform for solid tumors.

## Methods

### Cell lines and culture conditions

HEK293T (CRL-3216), Jurkat Clone E6-1 (TIB-152), HeLa (CCL-2), MDA-MB231 (HTB-26), HCT-116 (CCL-247), NALM6 (CRL3273) and MC38 (CRL-2640) were purchased from the American Type Culture Collection (ATCC, Rockville, MD). EGFR knockout HCT-116 (HCT-116^EGFR KO^) cells were purchased from Abcam (ab281597). NALM6^Luc^, HeLa^Luc^, MDA-MB231^Luc^, HCT-116^Luc^, and HCT-116^EGFR KO/Luc^ were generated in-house. The method for generation of firefly luciferase (Luc)-expressing cells have been described previously (19). HEK293T, HeLa, MDA-MB231, HCT-116 and MC38 cells were cultured in Dulbecco’s modified Eagle’s medium (DMEM) (#15400544, Lonza) supplemented with 2 mM L-glutamine (#11500626, Life Technologies), 10% (vol/vol) heat-inactivated fetal bovine serum (FBS, #F7524) and antibiotics (100 units/mL penicillin, 100 µg/mL streptomycin, #11548876) (both from Sigma-Aldrich), referred to as DMEM complete medium (DMC). NALM6 and Jurkat (Clone E6-1) cells were cultured in RPMI-1640 (#R0883, Lonza) supplemented with 2 mM L-glutamine, 10% heat-inactivated FBS and antibiotics, referred to as RPMI complete medium (RCM). All the cell lines were grown at 37 °C in 5% CO2 and routinely screened for mycoplasma contamination by PCR using the Mycoplasma Gel Detection Kit (#4542, Biotools).

### mRNA construct design and transfection

A capped and polyadenylated (120A) mRNA encoding an anti-EGFR x anti-CD3 bispecific antibody in light T cell engager (LiTE) format (18) was synthesized, using wild type bases, by TriLink BioTechnologies. The mRNA construct comprises the oncostain M signal peptide, an anti-human EGFR VHH Single Domain Antibody (EgA1), a five-residue linker (G_4_S), the CD3-specific OKT3 scFv (VH–VL) (20) and C-terminal myc/6His tags.

For transfection of HEK 293T cells, TransIT^®^-mRNA Transfection Kit (# MIR2250, Mirus Bio LLC) was used following manufacturer’s instructions. 48h after transfection, EGFR-LiTE transfected cells were analyzed for transfection efficacy by flow cytometry, as described below. Non-transfected (MOCK) HEK 293T cells were used as controls. In addition, culture supernatants from MOCK or EGFR-LiTE transfected cells were collected, stored at -20°C and subsequently used for Jurkat T cell activation assays.

For experiments with human primary T cells, peripheral blood mononuclear cells (PBMC) were isolated, by density gradient centrifugation using lymphoprep (#AXS-1114544, Cosmo-Bio), from peripheral blood of volunteer healthy donors or obtained from Buffy coats, provided by Madrid Blood Transfusion Center. All donors provided written informed consent in accordance with the Declaration of Helsinki. The study was approved by the Ethics Committee of Instituto de Investigación Hospital 12 de Octubre (Ethical approval number TP20/0094). Isolated PBMCs were activated with plate-bound 0.5 μg/mL anti-CD3 (OKT3; #566685) and 2μg/mL soluble anti-CD28 (CD28.2; #555725) antibodies (both from BD Bioscience) for 2 days. T cells were further expanded for 3–4 days in RCM supplemented with 100U/mL recombinant human IL-2 (rhIL-2, #703892.4O, Proleukin^®^, Clinigen). Then, T cells were washed and resuspended in OPTI-MEM (#10149832, Gibco) at a final concentration of 100x10^6^ cells/mL. Subsequently, the cells were mixed with 10 μg of EGFR-LiTE encoding mRNA per 10^6^ cells and electroporated in 4mm cuvettes (#1652081, BioRad), with a single 10 ms square-wave pulse of 250V, using the Gene Pulser Xcell Electroporation System (BioRad). As controls, T cells were left non-electroporated (NONe) or were electroporated without mRNA (mock-electroporated, MOCKe). Cells were maintained in RCM supplemented with 50U/mL of interleukin 2. Viability and electroporation efficiency (EGFR-LiTE expression) were analyzed at 3, 7, 24, 48, 72 and 96h hours post-electroporation by flow cytometry. For *in vivo* experiments, cells were aliquoted 3 hours after electroporation and cryopreserved at −80°C until use.

### Flow cytometry

Cell surface-bound EGFR-LiTEs were detected with APC-conjugated anti-His mAb (clone GG11-8F3.5.1, #130-119-782, Miltenyi Biotec). Intracellular EGFR-LiTE was detected using Inside Stain Kit (#130-090-477, Miltenyi Biotec), following the manufacturer’s instructions, and anti-His-APC mAb. For phenotypic analysis, the following antibodies were used: anti-human CD3-APC (clone UCHT-1, #555335, BD Biosciences), anti-human CD4-PE (clone L200, #555347, BD Biosciences), anti-human CD8-PE-Cy7 (clone RPA-T8, #560662, BD Biosciences), anti-human CD69-PE-Cy7 (clone L78, #560819, BD Biosciences), anti-human PD-1/CD279-FITC (clone MIH4, #557860, BD Biosciences), anti-human CD25-PE (clone M-A251, #555432, BD Biosciences), anti-human CD2-BV421 (clone RPA-2.10, #3000230, BioLegend), and anti-human CD3-V450 (clone UCHT-1, #560366, BD Biosciences). For activation assays, PE-conjugated anti-CD69 (clone L78, #341652, BD Biosciences), PeCy7-conjugated anti-CD3 (clone UCHT-1, #563423, BD Biosciences) and V450-conjugated anti-CD2 (clone S5.2, #644485, BD Biosciences) were used. 7-Aminoactinomycin D (7-AAD; #559925, BD Biosciences) was used as viability marker. For human EGFR expression analysis, BV421-conjugated anti-EGFR (EGFR.1, #566254, BD Biosciences) was used. For murine EGFR expression analysis, unconjugated anti-mouse EGFR (EGFR1, #ab30, Abcam) and secondary R-Phycoerythrin AffiniPure™ Goat Anti-Human IgG, Fcγ fragment specific (#109-115-190, Jackson ImmunoResearch) were used. Flow cytometry was performed using a CytoFLEX (Beckman Coulter) cytometer. Analysis was performed using FlowJo V10 software (Tree Star).

The gating strategies applied in flow cytometry analyses are summarized in **Supplementary Figure 1**.

### Activation assays

Non-electroporated (NONe), mock-electroporated (MOCKe) or EGFR-LiTE mRNA-electroporated (STAR^EGFR^)-T cells were allowed to recover for 3h, 24h, 48h, 72h and 96h after electroporation and then co-incubated with 5 x 10^4^ EGFR^-^ (NALM6 or HCT-116^EGFR KO^) or EGFR^+^ (HeLa, MDA-MB-231 or HCT-116) cells at 2:1 effector to target (E:T) ratio in U-bottom 96-well plates. After 24h, T cell activation was evaluated by staining with CD69, CD3 and CD2 and flow cytometry analysis. Additionally, supernatants from MOCK and EGFR-LiTE transfected HEK 293T cells, or from NONe-, MOCKe- and STAR^EGFR^-T cells, collected 7h, 24h and 48h after electroporation, were added to co-cultures of 1 x 10^5^ Jurkat cells with NALM6, HeLa, MDA-MB231, HCT-116 or HCT-116^EGFR KO^. After 24h, CD69 expression was analyzed by flow cytometry. MC38 cells were co-cultured with freshly isolated T cells in the presence of supernatants from MOCKe- and STAR^EGFR^-T cell cultures, collected 3, 24 and 48 post-electroporation. CD69 expression was evaluated 24h later.

### Cytotoxicity assays

For cytotoxicity assays, 1x10^5^ NONe-, MOCKe or STAR^EGFR^-T cells, collected at the indicated times post-electroporation, were co-cultured with luciferase-expressing EGFR^+^ (HeLa^Luc^, MDA-MB231^Luc^ or HCT-116^Luc^) or EGFR^-^ (NALM6^Luc^ or HCT-116^EGFR KO/Luc^) target cells at 2:1 E:T ratio. As controls, target cells were cultured in the absence of T cells. After 48 hours, supernatants were collected and stored at -20 °C for IFNγ secretion analysis, and 20 μg/mL D-luciferin (#E1602, Promega) was added before bioluminescence quantification using a Victor luminometer (PerkinElmer). Percent tumor cell viability was calculated as the mean bioluminescence of each sample divided by the mean of MOCKe-target cell samples x 100. Specific lysis was established as 100%-cell viability.

### Western blotting

To detect EGFR-LiTE secreted into the culture supernatant by transduced HEK293T cells, samples were separated under reducing conditions on 10% SDS-PAGE gels, transferred onto PVDF membranes (#OPVH00010, Merck Milipore) and probed with anti-His mAb (#34650, Qiagen; 200 ng/mL), followed by incubation with horseradish peroxidase (HRP)–conjugated goat anti-mouse (GAM) IgG, Fc specific (#12-349; Sigma Aldrich). Visualization of protein bands was performed with Pierce ECL Western Blotting substrate (#32134, Thermo Fisher Scientific).

### EGFR-LiTE and IFNγ detection by enzyme-linked immunosorbent assay

To detect EGFR-LiTE secreted into cell culture medium, recombinant human EGFR/Fc chimera (rhEGFR/Fc; #344-ER, R&D Systems) was immobilized (2.5 μg/mL) on Maxisorp plates (#M9410-1CS, Thermo Fisher Scientific) overnight at 4°C. After washing and blocking with 5% BSA in PBS, cell culture supernatants, collected at the indicated times post-electroporation, were added and incubated for 1h at room temperature. Then, wells were washed 3 times with PBS-0.05% Tween20 (#P1379, Sigma Aldrich) and 3 times with PBS (#508002, Werfen), and anti-His mAb (#34660, Qiagen) was added (1μg/mL). After washing, HRP-GAM IgG, Fc specific (1:2000 dilution; Sigma Aldrich) was added and the plate was developed using tetramethylbenzidine (TMB; #T0440, Sigma-Aldrich).

IFNγ secretion was analyzed by ELISA (#950.000.096, Diaclone), following the manufacturer’s instructions.

### *In vivo* studies

Animal procedures were adhered to European Union Directive 2010/63/UE, enforced in Spanish law under RD 53/2013. All animal experiments were approved by the respective Ethics Committee of Animal Experimentation of the Instituto de Investigación Hospital 12 de Octubre and Centro Nacional de Investigaciones Oncológicas (CNIO); they were performed in accordance with the guidelines stated in the International Guiding Principles for Biomedical Research Involving Animals, established by the Council for International Organizations of Medical Sciences. The experimental study protocols were additionally approved by local government (PROEX 166/19 and PROEX 272.2/23). *In vivo* experiments were conducted at the animal facilities of Hospital 12 de Octubre (2023) and in the Centro Nacional de Investigaciones Oncológicas (CNIO) (2024). All mice were housed under specific pathogen-free conditions, maintained on a 12-hour light/dark cycle and provided with sterile food and water ad libitum. For bioluminescence imaging, mice were anesthetized with 2% isoflurane in oxygen (flow rate: 1.5 L/min) and maintained at 1–1.5% during imaging. At the study endpoint, mice were euthanized by CO_2_ inhalation using a Vet-Tech euthanasia system at a displacement rate of 30–70% of the chamber volume per minute, and death was ensured. All euthanasia procedures were performed in accordance with the recommendations of the AVMA Guidelines for the Euthanasia of Animals.

9-week-old female NSG mice (NOD.Cg-Prkdc^scid^ Il2rg^tm1Wjl^/SzJ; The Jackson Laboratory) were injected subcutaneously with 5x10^6^ HeLa^Luc^ cells resuspended in 80% Matrigel basement membrane matrix (#356231, Corning). After four days, tumor volume was measured with caliper, and based on the tumor size, animals were homogeneously distributed in three groups to receive a first dose, via intratumoral (i.t.) injection, of vehicle (PBS), 15x10^6^ MOCKe-T cells or 15x10^6^ STAR^EGFR^-T cells. Three additional doses of 9x10^6^, 9x10^6^ and 8x10^6^ MOCKe-T or STAR^EGFR^-T cells were administered at days 7, 14 and 17 after first T cell infusion. Tumor volume was measured, and bioluminescence images were captured at the indicated time points to monitor tumor progression. For bioluminescence imaging, 125 mg/kg of D-luciferin (#E1605, Promega) was administered intraperitoneally. Animals were imaged 7 minutes after D-luciferase injection using the Bruker *In-Vivo* Xtreme II System (Bruker Corporation). The photon flux emitted by the luciferase-expressing cells was measured as an average radiance (P/s/mm^2). Imaging analysis was performed using the Bruker Molecular Imaging Software (Bruker). Mice were euthanized at day 25.

In another experiment, 9-week-old female NSG mice were injected subcutaneously with 5x10^6^ HeLa^Luc^ cells, followed by an intratumoral injection of vehicle (PBS), 10x10^6^ MOCKe-T cells or 10x10^6^ STAR^EGFR^-T cells at day 4. Three additional doses of 3x10^6^ MOCKe-T or STAR^EGFR^-T cells were administered at days 8, 11 and 14. Tumor growth and mice body weight were monitored every 3–4 days. Tumor volumes were calculated using the formula V = (D — d^2^)/2 mm^3^, where D is the largest diameter and d is the shortest diameter. Mice were euthanized at day 15. Tumor samples were excised and fixed (4% formalin solution) for histological examination (FFPE).

Finally, MDA-MB-231 cells (5x10^6^) were resuspended in 80% Matrigel basement membrane matrix (Corning) and implanted into right inguinal mammary fat pads of 9-week-old NSG female mice. When the tumors reached between 200–300 mm^2^, mice received four i.t. injections of vehicle (PBS), 10x10^6^ MOCKe-T cells or 10x10^6^ STAR^EGFR^-T cells on days 25, 28, 32 and 35. Tumor growth was monitored by caliper measurements twice a week. On day 36 two PBS-treated mice and three mice from MOCKe-T and STAR^EGFR^-T treatment groups were euthanized for collection of tumor, skin, lung, and liver samples for immunohistochemistry. Remaining mice were euthanized at day 39.

Body weight and clinical signs of disease, including graft-versus-host disease (GVHD), were regularly monitored in all the experiments. Humane endpoints were defined to proceed to euthanize if necessary.

### Immunohistochemistry

Tumors from different treatment groups were collected and fixed in 4% formalin solution (Sigma-Aldrich) for 48 hours and after extensive washing in PBS, tissues were embedded in paraffin. Four-µm-thick FFPE sections were processed on Dako PT Link pre-treatment system for optimized staining consistency. Antigen retrieval was performed with EDTA pH9 and sections were incubated with CD3 FLEX (#IR503, DAKO), CD8 FLEX (clone C8/144B, #IR623/IS623, DAKO) or Perforin (clone 5B10, #ab89821, Abcam) antibodies on Autostainer Link 48 (Dako) automated immunostaining platform. Nuclei were counterstained with Harris’ hematoxylin. Positive control sections known to be primary antibody positive were included for each staining run. All the slides were dehydrated, cleared and mounted with a permanent mounting medium for microscopic evaluation. Whole digital slides were acquired with a slide scanner (AxioScan Z1, Zeiss), and positive versus total cells were automatically quantified using QuPath v0.4.3 software (21).

### Statistical analysis

GraphPad Prism 8.0 was used to generate plots and to conduct statistical analyses. Results of experiments are presented as mean ± standard deviation (SD). Significant differences (P values) were identified using one-way or two-way analysis of variance (ANOVA) adjusted by Tukey’s test for situations involving multiple comparisons, as specified. Effect sizes were quantified as Cohen’s d, calculated from group means and pooled standard deviations. Confidence intervals for Cohen’s d (95% CI) were estimated using the noncentral t distribution following Hedges and Olkin. Statistical power (1-β) was computed for each comparison assuming α = 0.05, two-sided.

## Results

### Functional EGFR-LiTE is secreted by mRNA-transfected human cells

**Figure 1:**
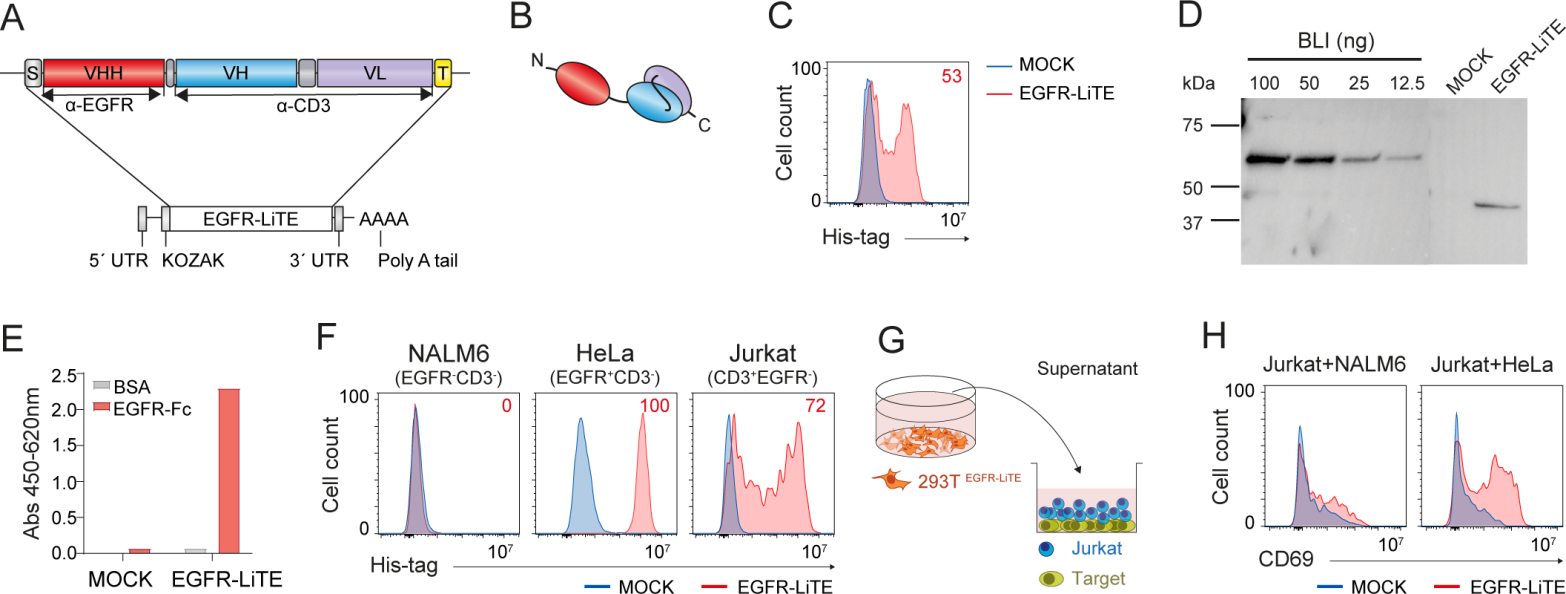
Functional mRNA-encoded EGFR-LiTE is efficiently secreted by human cells. **(A, B)** Schematic diagram showing the genetic **(A)** and domain **(B)** structure of anti-EGFR x anti-CD3 LiTE (EGFR-LiTE) bearing oncostatin M signal peptide (gray box), anti-EGFR VHH (EgA1) gene (red box), anti-CD3 scFv (OKT3) gene (blue and purple boxes), and Myc and His tags (yellow box). **(C)** EGFR-LiTE expression by mRNA-EGFR-LiTE transduced 293T cells 48 hours after transfection, assessed by intracellular staining using an anti-His-tag antibody. mock-transfected 293T cells (blue line) were used as negative controls. One representative experiment out of three independent experiments is shown. **(D)** Western blot detection of secreted EGFR-LiTE into the culture supernatant from mRNA-transfected 293T cells. Supernatant from mock-transfected 293T cells and decreasing concentrations of purified blinatumomab (BLI; 55 kDa) were used as negative and positive controls, respectively. One representative experiment out of three independent experiments is shown. **(E)** Detection of secreted EGFR-LiTE in the supernatant, collected 48 hours post-transfection, from mock-transfected or EGFR-LiTE-transfected 293T cells by ELISA against plastic-immobilized EGFR-Fc. One of two experiments performed in duplicate is shown. **(F)** Binding of EGFR-LiTE to target antigens expressed on cell surface, analyzed by flow cytometry. NALM6 (EGFR^-^CD3^-^), HeLa (EGFR^+^ CD3^-^) and Jurkat (CD3^+^EGFR^-^) cells were incubated for 30min with supernatant from EGFR-LiTE transfected or mock-transfected 293T cells and bound LiTE was detected using an anti-His-tag mAb. One representative experiment out of three independent experiments is shown. **(G)** Schematic representation of the co-culture system used to assess the ability of secreted EGFR-LiTE to recruit unmodified bystander T cells. **(H)** NALM6 (EGFR^-^) or HeLa (EGFR^+^) target cells (5x10^4^) were co-cultured with 1x10^5^ unmodified Jurkat cells in the presence of supernatant from EGFR-LiTE transfected or mock-transfected 293T cells. After 24 hours, CD69 expression by Jurkat T cells was evaluated by flow cytometry. One representative experiment out of three independent experiments is shown.

For this study we designed an mRNA encoding a bispecific anti-EGFR x anti-CD3 light T cell engager (EGFR-LiTE) (18) (**Figures 1A, B**). The EGFR-LiTE construct bears a 6xHis-tag for immunodetection (**Figure 1A**). To assess the ability of human cells to express EGFR-LiTE from *in vitro* transcribed (IVT) mRNA, we transfected 293T cells and analyzed the expression of the TCE by intracellular staining with an anti-His antibody (**Figure 1C**). EGFR-LiTE secreted by transfected 293T cells into the culture medium was detected by Western blot, showing the expected molecular weight (44kD) (**Figure 1D**), and specifically recognized both EGFR and CD3, either plastic-immobilized (**Figure 1E**) or expressed on the cell surface (**Figure 1F**). In Jurkat T cell activation experiments, significant CD69 upregulation occurred only when the T cell line was co-cultured with HeLa (EGFR^+^) tumor cells in the presence of EGFR-LiTE-transfected cell supernatant, but not when co-cultured with NALM6 (EGFR^-^) cells or in the presence of mock-transfected cell supernatant (**Figures 1G, H**), suggesting that the secreted TCE specifically mediates T cell activation against EGFR-positive tumor cells.

### mRNA-modified T cells efficiently secrete EGFR-LiTE and activate against EGFR^+^ tumor cells

**Figure 2:**
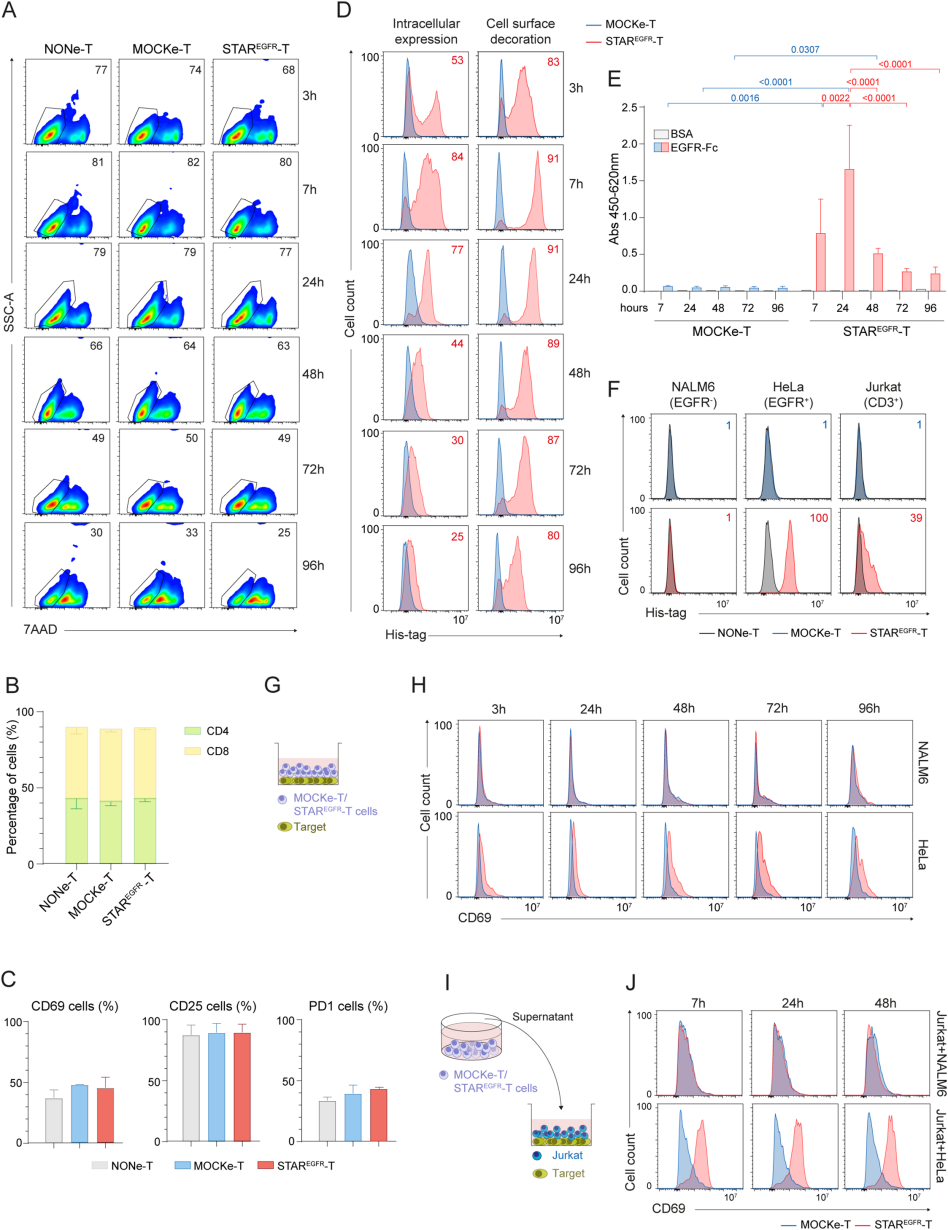
Engineering of functional STAR^EGFR^-T cells by electroporation of human primary T cells. Human primary T cells were expanded for 6 days and either left non-electroporated (NONe-T), electroporated in the absence of mRNA (mock-electroporated; MOCKe-T) or electroporated with EGFR-LiTE encoding mRNA (STAR^EGFR^-T). **(A)** Percentage of viable (7AAD^-^) NONe-, MOCKe- and STAR^EGFR^- T cells at different time points post-electroporation. One representative experiment of three independent experiments performed with T cells from different donors is shown. **(B, C)** Percentages of **(B)** CD4^+^ and CD8^+^ T cells and **(C)** of CD69^+^, CD25^+^ and PD1^+^ cells among NONe-, MOCKe- and STAR^EGFR-^T cells. Results are mean ± SD of three experiments performed with T cells from different donors. **(D)** Intracellular expression and cell-surface bound EGFR-LiTE (decoration) in STAR^EGFR^-T cells (pink line) at different time points after electroporation. MOCKe-T cells (blue line) were used as negative controls. One representative experiment of six independent experiments performed with T cells from different donors is shown. Descriptive statistics (mean, SD, CV) are provided in **Supplementary Table 1**. **(E)** Detection, by ELISA against plastic-immobilized EGFR-Fc, of soluble EGFR-LiTE in the supernatant from MOCKe-T and STAR^EGFR-^T cells, collected at different times post-electroporation. Data are expressed as mean ± SD of three independent experiments performed with T cells from different donors. Differences were analyzed by a two-way ANOVA test corrected with a Tukey´s multiple comparison test. Descriptive statistics (mean, SD, CV) are provided in **Supplementary Table 2**. **(F)** NALM6 (EGFR^-^CD3^-^), HeLa (EGFR^+^ CD3^-^) and Jurkat (CD3^+^EGFR^-^) cells were incubated for 30min with supernatant from STAR^EGFR^-, MOCKe- or NONe-T cells collected 24 hours post-electroporation. LiTE bound to target antigens expressed on cell surface was detected by flow cytometry using an anti-His-tag mAb. One representative experiment out of three independent experiments performed with T cells from different donors is shown. **(G, H)** MOCKe- or STAR^EGFR^-T cell were collected at different time points post-electroporation and co-cultured with NALM6 (EGFR^-^) or HeLa (EGFR^+^) cells at an effector:target ratio (E:T) 2:1 for 24 hours. **(G)** Schematic representation of the co-culture system; **(H)** CD69 expression on T cells was analyzed by flow cytometry. **(I, J)** Unmodified Jurkat T cells were co-cultured for 24 hours with NALM6 or HeLa target cells at 2:1 E:T ratio in the presence of supernatants from MOCKe-T or STAR^EGFR^-T cells collected at different time points. **(I)** Schematic representation of the co-culture system. **(J)** Expression of the CD69 activation marker was analyzed by flow cytometry. **(H, J)** show one representative experiment out of five performed with T cells from different donors.

Once demonstrated that the IVT mRNA can drive functional EGFR-LiTE secretion, we analyzed the ability of human primary T cells to produce the mRNA-encoded TCE and specifically target EGFR-expressing cells. For this purpose, PBMCs from healthy donors were stimulated with anti-CD3, anti-CD28 and IL-2, and electroporated after 6 days with EGFR-LiTE-encoding mRNA to generate STAR^EGFR^-T lymphocytes. Their viability at different time points was similar to that of non-electroporated (NONe)-T cells and of cells electroporated without mRNA (MOCKe-T cells) (**Figure 2A**, **Supplementary Figure S2**). Similarly, a phenotypic analysis performed 24 hours post-electroporation showed no differences in the proportion of CD4^+^ and CD8^+^ cells, nor in the expression of molecules involved in T cell function such as CD25, CD69 and PD-1, between NONe, MOCKe and STAR^EGFR^-T cells (**Figures 2B, C**). STAR^EGFR^-T cells successfully produced functional TCE for several days, as observed by intracellular expression and positive surface staining (decoration) with anti-His-tag mAb (**Figure 2D**). The EGFR-LiTE secreted to the supernatant specifically bound plastic-immobilized human EGFR-Fc chimeric protein (**Figure 2E**) and recognized both of its targets, EGFR and CD3 expressed on the cell surface (**Figure 2F**).

To assess whether the secreted EGFR-LiTE could induce STAR-T cell activation in the presence of EGFR-expressing tumor cells, STAR-T lymphocytes were collected at different time points post-electroporation and co-cultured with NALM6 (EGFR^-^) or HeLa (EGFR^+^) tumor cells for 24 hours (**Figure 2G**). The expression of the activation marker CD69 was then evaluated. STAR-T lymphocytes were specifically activated only in the presence of cells expressing the target antigen, EGFR (**Figure 2H**). Despite the inherently transient nature of mRNA, EGFR-specific activation could be observed even 96 hours after the electroporation (**Figure 2H**) suggesting that although a smaller amount of EGFR-LiTE is being produced at that time point, it is enough for efficient T cell recruitment against target cells. In contrast, MOCKe-T cells or STAR-T cells co-cultured with EGFR-negative NALM6 did not show increased CD69 expression, beyond that derived from the activation protocol used for T cell expansion and/or some degree of alloreactivity against target cells (**Figure 2H**, **Supplementary Figures S3A, B**). To evaluate the potential of the TCE secreted by STAR-T cells into the culture medium to recruit non-modified T lymphocytes, co-cultures of Jurkat T cells with EGFR^+^ or EGFR^-^ target cells were performed in the presence of supernatant from STAR^EGFR^-T cells (**Figure 2I**). As expected, CD69 expression increased in Jurkat T cells co-cultured with HeLa (EGFR^+^) cells and conditioned medium from STAR^EGFR^-T cells collected at different time points, but not when co-cultured with NALM6 (EGFR^-^) target cells (**Figure 2J**). Supernatant from MOCKe-T cells did not have any effect on T cell activation (**Figure 2J**). These results suggest that EGFR-LiTEs secreted by STAR^EGFR^-T cells recruit non-modified T cells to engage and attack EGFR^+^ cells. Similar results were obtained when MDA-MB-231 or HCT-116 EGFR-expressing cell lines were used as targets in activation assays (**Supplementary Figures S4A-E**). In these experiments, HCT-116 ^EGFR KO^ cells served as a negative control (**Supplementary Figures S4D, E**).

### STAR^EGFR^-T cells exert cytotoxic activity against EGFR^+^ cell lines *in vitro*

**Figure 3:**
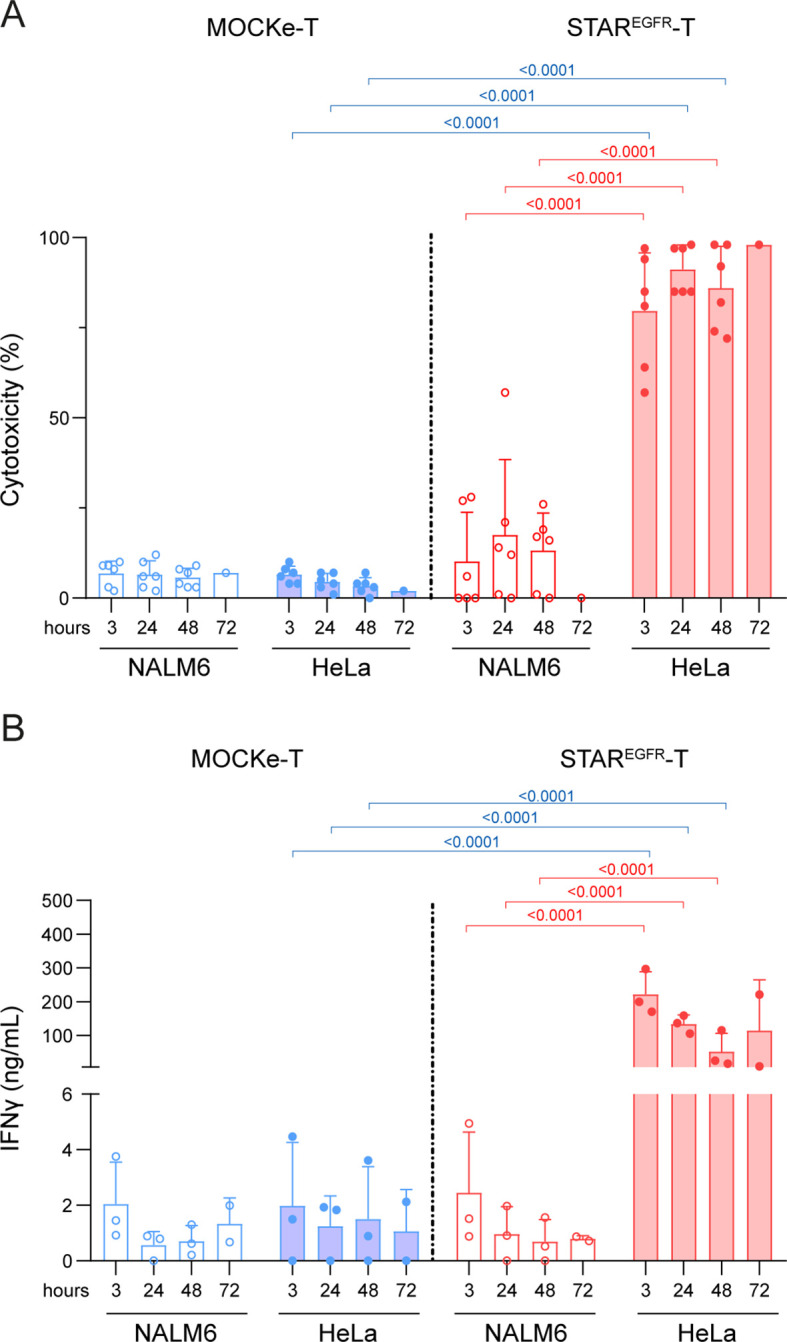
STAR^EGFR^-T cells show cytotoxic activity against EGFR^+^ tumor cells *in vitro.* MOCKe- or STAR^EGFR^-T cells (1x10^5^) were collected at different post-electroporation time points and co-cultured with 5x10^4^ NALM6^Luc^ (EGFR^-^) or HeLa^Luc^ (EGFR^+^) cells. After 48 hours, **(A)** the percentage of specific cytotoxicity was calculated by adding D-luciferin to detect bioluminescence, and **(B)** the level of IFNγ secretion was determined by ELISA. Data represent mean ± SD of six independent experiments performed in triplicate with T cells obtained from six different donors. Significance was calculated by a two-way ANOVA test corrected with a Tukey´s multiple comparisons test. Descriptive statistics (mean, SD, CV) are provided in **Supplementary Tables 3** and **4**. ANOVA, analysis of variance; MOCKe-T, mock-electroporated; STAR^EGFR^-T, EGFR-LiTE electroporated.

To assess the cytotoxic activity of STAR^EGFR^-T lymphocytes, electroporated T cells were co-cultured with luciferase-expressing target cells (HeLa^Luc^ and NALM6^Luc^). After 48 hours, D-luciferin was added, and bioluminescence was measured. STAR^EGFR^-T cells induced almost 100% lysis of EGFR-positive tumor cells, and their cytotoxic capacity did not decline even 72 hours post-electroporation (**Figure 3A**). As expected, cytotoxic activity against EGFR^-^ target cells, and MOCKe-T cell mediated lysis were marginal (**Figure 3A**). Similar results were obtained in the IFNγ secretion analysis (**Figure 3B**). To further confirm these observations, STAR^EGFR-^T cytotoxicity and IFNγ secretion were evaluated in co-cultures with MDA-MB231^Luc^ and HCT-116^Luc^ EGFR-expressing cell lines (**Supplementary Figures S4F, G**), using HCT116^EGFR KO/Luc^ cells as EGFR-negative control. Tumor cell killing and IFNγ secretion did not correlate with the EGFR expression levels of the different cell lines (**Supplementary Figures S4F-I**).

### STAR-T cells control the growth of EGFR^+^ tumors *in vivo*

**Figure 4:**
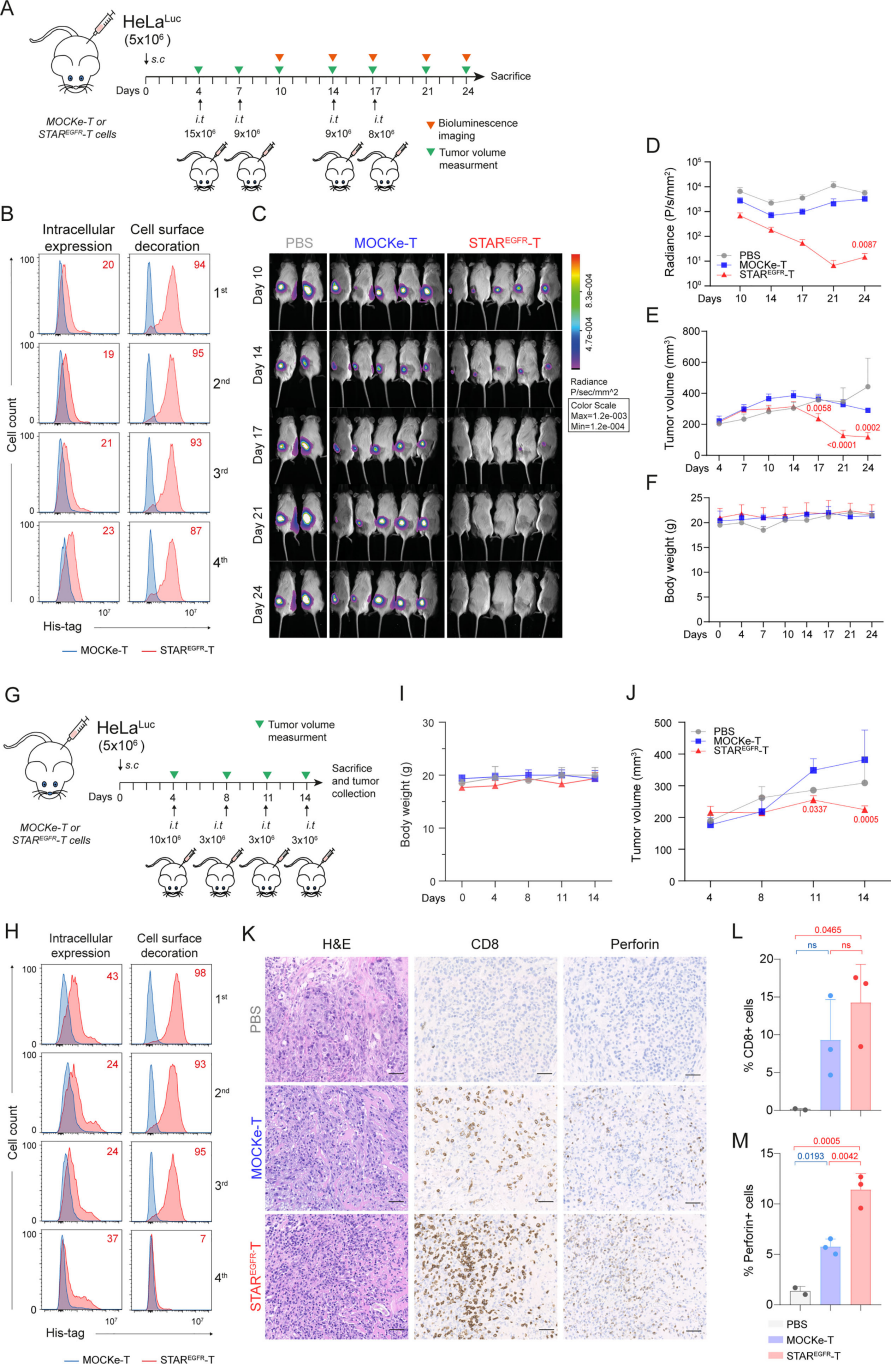
STAR^EGFR^-T cells control EGFR^+^ tumor growth *in vivo.***(A)** Experimental design of high-dose HeLa xenograft murine model. NSG mice were subcutaneously (s.c.) injected with 5x10^6^ HeLa^Luc^ cells followed by 4 intratumoral (i.t.) administrations of 15, 9, 9 and 8x10^6^ MOCKe-T (n=5) or STAR^EGFR^-T cells (n=5) on days 4, 7, 14 and 17 after tumor implantation, respectively. Mice injected with an equal volume of PBS (n=2) were used as controls. **(B)** Upon thawing, a few MOCKe- or STAR^EGFR^-T cells were left to recover in culture for 24 hours and intracellular expression and surface-bound EGFR-LiTE (decoration) were analyzed by flow cytometry (n=1). **(C)** Bioluminescence images monitoring tumor progression. **(D)** log10 of total radiance quantification at the indicated time points. **(E)** Tumor volume (mm^3^) measured by caliper in the different treatment groups over time. **(F)** Body weight of mice at the indicated time points. In E and F, PBS, n=2; MOCKe-T, n=5; STAR^EGF^R-T, n=5. Data are expressed as mean ± SD. Differences were analyzed by a two-way ANOVA test corrected with a Tukey´s multiple comparison test. Effect size and *post hoc* power were calculated for radiance and tumor volume at day 24: Radiance, Cohen’s d = 2.02, 95% CI = [0.43, 3.61], (1–β) > 0.99 (α = 0.05, two-sided); Tumor volume, Cohen’s d = 4.67, 95% CI = [2.07, 7.28], (1–β) > 0.99 (α = 0.05, two-sided). **(G)** Experimental design of low-dose HeLa xenograft model. NSG mice were s.c. injected with 5x10^6^ HeLa^Luc^ cells followed by 4 i.t. administrations of 10, 3, 3 and 3x10^6^ MOCKe-T (n=3) or STAR^EGFR^-T (n=3) cells on days 4, 8, 11 and 14 after tumor implantation, respectively. Mice injected with an equal volume of PBS (n=2) were used as controls. **(H)** Upon thawing, a few engineered MOCKe- or STAR^EGFR^-T cells were left to recover in culture for 24 hours and intracellular expression and surface-bound EGFR-LiTE (decoration) was analyzed by flow cytometry (n=1). **(I)** Body weight and **(J)** tumor volume (mm^3^) measured by caliper in the different treatment groups over time (PBS, n=2; MOCKe-T, n=3; STAR^EGF^R-T, n=3). **(K)** Hematoxylin and eosin, CD8 and perforin staining of representative tumor sections from mice treated with PBS, MOCKe-T of STAR^EGFR^-T cells. **(L, M)** Quantification of CD8^+^**(L)** and perforin^+^**(M)** cells in the tumor sections from PBS (n=2), MOCKe-T (n=3) and STAR^EGFR^-T (n=3) treated animals. Data are expressed as mean ± SD. Differences were analyzed by a two-way ANOVA test corrected with a Tukey´s multiple comparison test. Effect size and *post hoc* power were calculated for tumor volume at day 14 and for the percentage of perforin-positive cells: Tumor volume, Cohen’s d = 2.37, 95% CI = [0.08, 4.66], (1–β) > 0.85 (α = 0.05, two-sided); % Perforin^+^ cells: Cohen’s d = 4.5, 95% CI = [1.34, 7.66], (1–β) > 0.99 (α = 0.05, two-sided).

**Figure 5:**
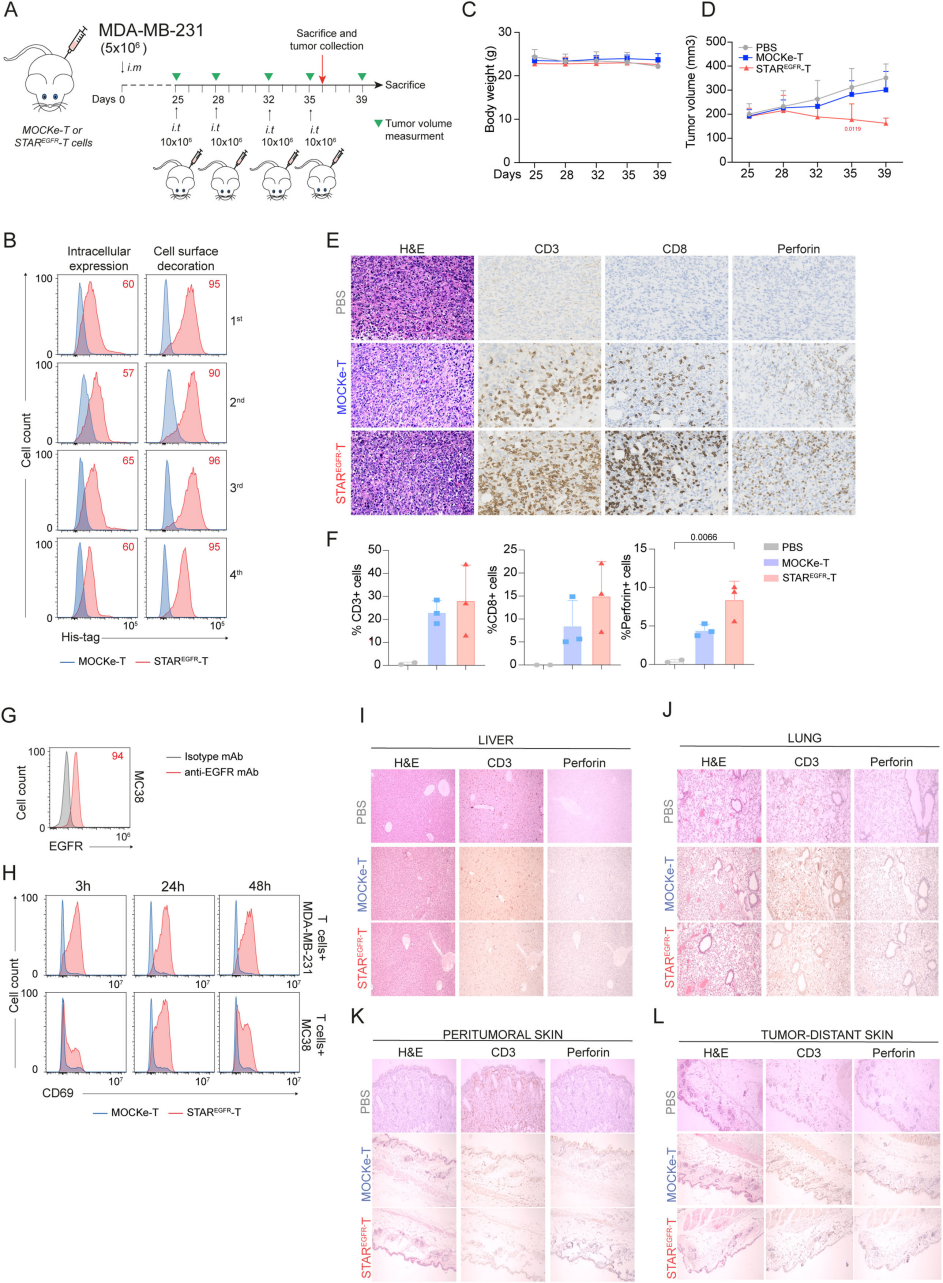
STAR^EGFR^-T cell anti-tumor effect is not associated with on-target/off-tumor toxicity in an *in vivo* model. **(A)** Experimental design of MDA-MB-231 breast cancer orthotopic murine model. NSG mice were intramammary (i.m.) injected with 5x10^6^ MDA-MB231 cells followed by 4 intratumoral (i.t.) administrations of 10x10^6^ MOCKe-T (n=8) or STAR^EGFR^-T cells (n=8) on days 4, 7, 14 and 17 after tumor implantation. Mice injected with an equal volume of PBS (n=4) were used as controls. **(B)** Upon thawing, a few MOCKe- or STAR^EGFR^-T cells were left to recover in culture for 24 hours and intracellular expression and surface-bound EGFR-LiTE (decoration) were analyzed by flow cytometry (n=1). **(C)** Body weight and **(D)** tumor volume (mm^3^) measured by caliper in the different treatment groups over time (PBS, n=4; MOCKe-T, n=8; STAR^EGF^R-T, n=8 for days 25-35; PBS, n=2; MOCKe-T, n=5; STAR^EGF^R-T, n=5 for day 39). Data are expressed as mean ± SD. Differences were analyzed by a two-way ANOVA test corrected with a Tukey´s multiple comparison test. Effect size and *post hoc* power were calculated for tumor volume at day 35 and for the percentage of perforin-positive cells: Tumor volume, Cohen’s d = 1.70, 95% CI = [0.54, 2.87], (1–β) > 0.99 (α = 0.05, two-sided); % Perforin^+^ cells: Cohen’s d = 2.17, 95% CI = [0.05, 4.29], (1–β) > 0.80 (α = 0.05, two-sided). **(E)** Hematoxylin and eosin, CD3, CD8 and perforin staining of representative tumor sections from mice treated with PBS (n=2), MOCKe-T (n=3) of STAR^EGFR^-T (n=3) cells. **(F)** Quantification of CD3^+^, CD8^+^ and perforin^+^ cells in the tumor sections from PBS (n=2), MOCKe-T (n=3) and STAR^EGFR^-T (n=3) treated animals. Data are expressed as mean ± SD. Differences were analyzed by a two-way ANOVA test corrected with a Tukey´s multiple comparison test. **(G)** Analysis of EGFR expression by MC38 murine cells. Representative result from three independent staining experiments. **(H)** Unmodified human primary T cells were co-cultured with human MDA-MB-231 or murine MC38 cells at 2:1 E:T ratio, in the presence of supernatants from MOCKe-T or STAR^EGFR^-T cells collected at different time points. After 24h, expression of CD69 was analyzed by flow cytometry. One representative experiment out of three independent experiments is shown. **(I-L)** Hematoxylin-eosin, CD3 and perforin staining was performed in samples collected from liver **(I)**, lung **(J)** and skin, both peritumoral **(K)** and tumor-distant **(L)** skin. Representative images of tumor sections from mice treated with PBS (n=2), MOCKe-T (n=3) of STAR^EGFR^-T (n=3) cells.

To study the antitumor effect of STAR^EGFR^-T cells *in vivo*, 5x10^6^ HeLa^LUC^ cells were injected subcutaneously (s.c.) into the right flank of NSG mice. Four intratumoral (i.t.) administrations of 15, 9, 9 and 8x10^6^ MOCKe-T or STAR^EGFR^-T cells were performed on days 4, 7, 14 and 17, after tumor implantation, respectively (**Figure 4A**). T cells derived from a single batch of electroporated cells, which were subsequently aliquoted and cryopreserved, were used for all infusions. TCE expression and viability of the freshly electroporated T cells are shown in **Supplementary Figures S5A, B**. After each infusion, a sample of thawed T cells was cultured for 24 hours and analyzed for viability and EGFR-LiTE expression. In all the cases, cell viability of both STAR^EGFR^-T and MOCKe-T thawed cells was similar, although, as expected, lower than that of cells before the freeze-thaw cycle (**Figure 5B**). Notably, STAR^EGFR^-T cells corresponding to each of the four infusions showed similar intracellular expression and cell surface decoration (**Figure 4B**). Mice receiving STAR^EGFR^-T cells showed significant tumor regression compared to MOCKe-T cell or PBS treated mice, as evidenced by bioluminescence (**Figures 4C, D**) and tumor volume determination (**Figure 4E**). No decrease in body weight was observed during the treatment (**Figure 4F**).

In order to perform histological analyses assessing the presence and activity of T cells within the tumor, we conducted an *in vivo* assay injecting lower doses of T cells, and mice were euthanized when they still had measurable tumors. Thus, 5x10^6^ HeLa^LUC^ cells were injected s.c. into the right flank of NSG mice and one dose of 10x10^6^ cells and three doses of 3x10^6^ MOCKe-T or STAR^EGFR^-cells were administered i.t. on days 4, 8, 11 and 14, respectively (**Figure 4G**). Viability and EGFR-LiTE expression over time, corresponding to freshly electroporated and to thawed cells for each infusion, are shown in **Supplementary Figure S6** and **Figure 4H**. Body weight did not change in any treatment group (**Figure 4I**). As expected, STAR^EGFR^-T cells exerted greater control over tumor growth than MOCKe-T cells but did not completely eliminate tumors (**Figure 4J**). This allowed us to perform histological analysis of tumor samples collected two weeks after treatment initiation, observing statistically significant higher numbers of T cells in STAR^EGFR^-T group compared to MOCKe-T treated group (**Figures 4K, L**). Moreover, we observed a significantly higher presence of perforin-positive cells in STAR^EGFR^-T-treated tumors, demonstrating that a potent cytotoxic anti-tumor response is in progress (**Figures 4K, M**).

The *in vivo* anti-tumor effect of STAR^EGFR^-T cells was further evaluated in an orthotopic model of breast cancer. 5x10^6^ MDA-MB-231 cells were injected into the mammary fat pads of NSG mice. Four i.t. administrations of PBS (n=4) or 10x10^6^ MOCKe-T (n=8) or STAR^EGFR^-T (n=8) cells, from a single batch of frozen cells, were performed on days 25, 28, 32 and 35 after tumor implantation (**Figure 5A**). The viability of electroporated cells and EGFR-LiTE expression, before freezing and after thawing, are shown in **Supplementary Figure S7** and **Figure 5B**. No changes in body weight were observed during the treatment (**Figure 5C**). **Figure 5D** shows tumor growth inhibition by STAR^EGFR^-T cells, compared to tumor progression in PBS- and MOCKe-T cell treated mice. One day after the fourth and final T cell injection, two PBS-treated mice and three mice from MOCKe-T and STAR^EGFR^-T treatment groups were euthanized and tumor, skin, lung and liver samples were collected. Immunohistochemistry analysis revealed an increased presence of CD3^+^ and CD8^+^ T cells and a significantly higher frequency of perforin-positive cells in tumors from STAR^EGFR^-T-treated mice, compared to those receiving MOCKe-T cells (**Figures 5E, F**). Finally, we evaluated the potential on-target/off-tumor toxicity of STAR^EGFR^-T cells in EGFR-expressing tissues other than the tumor. The EGFR-specific VHH Ega1 used to generate the EGFR-LiTE is a well-characterized antibody that recognizes both human and mouse EGFR (22). Indeed, the activation of human T cells against the EGFR^+^ mouse cell line MC38 (**Figure 5G**) in the presence of STAR^EGFR^-T cell supernatant was assessed *in vitro* (**Figure 5H**). This activation was EGFR-LITE dependent, as T cells were not activated when co-cultured with MC38 cells in the presence of MOCKe-T cell supernatant (**Figure 5H**). Samples from liver, lung and skin, both peritumoral and tumor-distant skin, were analyzed for T cell infiltration and activation (**Figures 5I-L**). Hematoxylin-eosin, CD3 and perforin staining revealed normal histology in all organs, with no differences between MOCKe- and STAR^EGFR^-T-treated mice, and no evidence of inflammatory infiltrate. Therefore, no detectable signs of on-target/off-tumor toxicity was observed following intratumoral administration of STAR^EGFR^-T cells transiently secreting an anti- EGFR-TCE.

## Discussion

Adoptive cell therapy with CAR-T cells has shown potent antitumor responses in hematologic malignancies. However, they have had limited success in solid tumors, due to significant challenges, such as trafficking and infiltration into the tumor and/or overcoming the strongly immunosuppressive microenvironment (23). In addition, the difficulty in identifying truly cancer-specific antigens leads to on-target/off-tumor toxicity, due to the attack of healthy tissues that express the target antigen (24, 25). The permanent integration of the CAR transgene into the genome, achieved by viral transduction, can turn this toxicity into long-lasting. In recent years, T cell modification with mRNA has emerged as a safer, rapid and cost-effective alternative to engineering with viral vectors, and mRNA-CAR-T cells are being evaluated in clinical trials.

On the other hand, we and others have recently reported the superior efficacy of TCE-secreting STAb-T cells over CAR-T lymphocytes as a strategy to redirect T cell responses against hematological tumors (1–4). In this study we report the generation of mRNA-engineered TCE-secreting STAR-T cells directed against solid tumors. Specifically, human primary T cells were electroporated with an mRNA encoding an EGFR x CD3 LiTE. The secreted soluble EGFR-LiTE successfully redirected T cells to bind EGFR on tumor cell surface, converting them into efficient tumor cell killers.

Significant T cell-surface decoration with EGFR-LiTE persisted for 4 days *in vitro*, indicating that, despite the transient nature of mRNA, engineered T lymphocytes remain armored to attack cancer cells for several days. However, transgene expression dropped after 96 hours. This expression period is similar to that observed in other studies with mRNA-engineered CAR-T cells (26, 27), although slight variations, based on differences in IVT vector and modifications in RNA structure, have been reported (27). In addition, differences in pre- and post-electroporation T cell expansion protocols might influence the efficiency of electroporation and the persistence of transgene expression.

In accordance with decoration levels, STAR^EGFR^-T cells showed ability to exert specific cytotoxicity against EGFR^+^ tumor cells up to 72 hours after electroporation. Interestingly, STAR-T cell cytotoxic efficiency did not directly correlate with the level of EGFR expression. This observation is consistent with previous data on anti-EGFRvIII CAR-T cells secreting anti-EGFR-TCEs, which reduced viability of multiple EGFR^+^ EGFRvIII^-^ glioma cell lines in a way that was not clearly related to the level of EGFR expression (5). Differential expression of other molecules that modulate the immune response could explain this phenomenon.

Corroborating the *in vitro* results, STAR^EGFR^-T cells efficiently abrogated tumor growth *in vivo*. Mouse xenograft models of leukemia have been successfully treated with systemically administered mRNA-CAR-T cells (13). However, most studies analyzing mRNA-CAR-T efficacy in solid tumors have been reported performing local or intratumoral injection of the cells (13), presumably to avoid difficulties penetrating the tumor. Thus, anti-human mesothelin CAR-T cells showed antitumor activity when injected i.p. but not i.v (28). Therefore, in this proof-of-concept study we have performed intratumoral administration of STAR-T cells. Concerns may arise regarding the clinical translatability of our results, given the use of immunodeficient xenograft models and the reliance on intratumoral injection. Although immunodeficient models lack a functional immune system and do not fully recapitulate the tumor microenvironment, they still provide essential proof-of-concept data directly supporting clinical translation, and have underpinned the approval of transformative immunotherapies such as tisagenlecleucel and (29) or idecabtagene vicleucel (30). With respect to intratumoral administration, while not universally applicable, biopsies are routinely performed in many solid tumors and could be leveraged for local therapeutic delivery. Moreover, implantable reservoirs, already used in oncology (e.g., Ommaya reservoirs, hepatic infusion pumps, intraperitoneal catheters) could enable repeated dosing. Nevertheless, evaluation in immunocompetent models remains an important consideration, and systemic administration continues to be a major objective. In this regard, previous data from our group suggest efficient trafficking of systemically administered T cells stably secreting anti-EGFR TCEs (31). On the other hand, it is worth noting that local administration of the therapy may not only promote treatment efficacy but also further reduce systemic toxicity. Treatment with anti-EGFR antibodies is particularly associated with dermatologic toxicities, due to the widespread expression of EGFR in epithelial tissues (32). Importantly, we have not observed on-target/off-tumor toxicities even when EGFR-LiTE is able to mediate T cell activation against murine EGFR. Interestingly, local administration of EGFRvIII-targeting CAR-T cells that simultaneously secrete TCEs against wild-type EGFR, achieved radiographic tumor regression in patients with glioblastoma, and demonstrated a safe profile (7).

A major concern is the potential generation of anti-TCE antibodies, especially in the context of a repeated dose scheme. In this regard, development of anti-murine CAR antibodies has been reported in clinical trials with mRNA-CAR-T cells, resulting in anaphylactic reactions (12, 33). For that reason, the use of humanized or fully human scFv has been proposed (12). For the clinical application of STAR-T therapy, we are developing humanized anti-EGFR VHH and anti-CD3 scFv candidates.

In solid tumors, homing and penetration of adoptively transferred T cells is hampered by barriers imposed by the tumor microenvironment, including a limited vascular system, increased interstitial pressure and a dense extracellular matrix (34, 35). In this scenario, small TCEs secreted by STAR-T cells reaching the periphery of the tumors might penetrate and recruit T lymphocytes present in the tumor bed, boosting their activity. This might represent an advantage of STAR-T lymphocytes over CAR-T cells, whose effector molecule is membrane-anchored.

On the other hand, clinical evidence correlates the efficacy of CAR-T therapy with the long-term persistence of CAR-T cells in the patient (23, 36). Thus, in mRNA-based therapies, the short duration of mRNA expression might mitigate off-target effects but could also reduce the chance of long-term remission. This limitation of transient mRNA-based T cell modification may be overcome by performing repeated administrations. In fact, clinical trials using mRNA-CAR-T cells for both hematological and solid tumors have shown that multiple infusions are necessary to prolong the therapeutic effect (13). Consequently, we have provided four infusions of STAR-T cells to fully control tumor growth. In this regard, a potential shortcoming could be the challenge of generating enough mRNA-engineered T cells for the required doses to ensure successful treatment of the patient. Thus, the impossibility of generating the planned doses have been reported in a clinical trial using CD123-specific mRNA-CAR-T cells. However, our previous studies comparing lentivirally transduced STAb-T versus CAR-T cells have shown the superior potency of TCE-secreting lymphocytes due to polyclonal recruitment of unmodified bystander T cells (1, 4). This implies that considerably fewer STAb-T cells are required to achieve the same antitumor effect, which may compensate for the difficulty in obtaining high numbers of mRNA-modified T cells.

As an alternative to *in situ* secretion by modified T cells, systemic delivery of TCE-encoding mRNA, most often formulated with lipid or polymeric nanoparticles, is emerging as an off-the-shelf approach. Preclinical studies have demonstrated both safety and antitumor activity (37–40) and a first-in-human trial is ongoing in patients with solid tumors (41). While this approach simplifies manufacturing and broadens patient applicability, it lacks the advantage of T cells serving as both antibody factories and tumor-homing effectors. Targeted nanoparticles that transiently modify T cells *in vivo* (42) may integrate both benefits, though efficiency and uniformity of *in vivo* modification remain challenging.

Another strategy to mitigate on-target/off-tumor toxicity is the administration of protease-activated TCEs, such as Probodies (43) or precision-activated TCEs (XPATs) (44). In these molecules, antibody-binding domains are masked and become active only within the protease-rich tumor microenvironment (45). Masked TCEs targeting different tumor antigens, including EGFR, retained potent antitumor efficacy while markedly reduced toxicity *in vivo*, and are progressing into clinical trials (45). Compared with the STAR-T approach, masked TCEs offer the advantage of being off-the-shelf therapies, but raise concerns about tumor homing, incomplete activation or potential immunogenicity.

Finally, immunotherapies targeting multiple antigens can reduce the risk of immune escape caused by antigen loss. Dual-targeting approaches that combine a CAR with a secreted TCE may further enhance efficacy by recruiting bystander T cells, while also benefiting from CAR-mediated costimulatory signals, potentially supporting long-term persistence. Virally transduced CAR-BiTE/CAR-STAb-T cells have shown encouraging results in preclinical models of hematological (46) and solid tumors (5, 6), and have entered clinical trials (7). Moreover, mRNA-electroporated CAR–HLA-G γδ T cells secreting an anti–PD-L1 × anti-CD3 TCE have demonstrated efficacy against solid tumors *in vivo* (17). Here, we show for the first time that STAR-T cells, in the absence of CAR expression, can reduce solid tumor growth, while our platform also remains compatible with dual-targeting strategies combining CARs and/or additional TCEs.

In summary, this study supports the potential of mRNA-engineered T cells secreting TCEs against an overexpressed antigen in epithelial tumors as a therapeutic approach for solid tumor patients, which may reduce the on-target/off-tumor toxicity associated with current T cell-redirecting therapies.

## Data Availability

The raw data supporting the conclusions of this article will be made available by the authors, without undue reservation.

## Glossary

7-AAD: 7-Aminoactinomycin D
ATCC: American Type Culture Collection
B-ALL: B cell Acute Lymphoblastic Leukemia
CAR: Chimeric antigen receptor
DMEM: Dulbecco’s Modified Eagle’s Medium
DMC: DMEM complete medium
ECL: Enhanced chemiluminescence
EGFR: Epidermal Growth Factor Receptor
ELISA: Enzyme-linked immunosorbent assay
E:T: Effector:target ratio
Fig.: Figure
FBS: Fetal bovine serum
FFPE: Formalin-Fixed Paraffin-Embedded
GAM: Goat anti-mouse
HRP: Horseradish peroxidase
IFNγ: Interferon gamma
IL: Interleukin
ITV: *In vitro* transcribed
I.V.: Intravenous
I.T.: Intratumoral
LiTE: Light T-cell engager
LUC: Luciferase
mAb: Monoclonal antibody
mRNA: Messenger ribonucleic acid
NOD: Non-obese diabetic
NSG: NOD.Cγ-Prkdcscid Il2rgtm1Wjl/SzJ mice
NONe: Non-electroporated
MOCKe: Mock-electroporated
MOCK: Mock-transfected
PB: Peripheral blood
PBS: Phosphate-buffered saline
PBMC: Peripheral blood mononuclear cells
PVDF: Polyvinylidene Fluoride
RPMI: Roswell Park Memorial Institute
RCM: RPMI complete medium
RH: Recombinant human
scFv: Single-chain Fragment variable
S.C.: Subcutaneous
SD: Standard deviation
SDS-PAGE: Sodium dodecyl-sulfate polyacrylamide gel electrophoresis
STAb: Secreting T cell-engaging antibodies
STAR: STAb-T cells engineered with mRNA
TCE: T cell engager
TMB: 3’,5,5’-tetramethylbenzidine
VHH: Single-domain antibodies from camelid heavy-chain-only immunoglobulins
