# Supplementary material for: mRNA-engineered T lymphocytes secreting bispecific T cell engagers with therapeutic potential in solid tumors
Source: Front Immunol. 2025 Nov 18;16:1684655. doi: 10.3389/fimmu.2025.1684655 (PMC12669218; doi:10.3389/fimmu.2025.1684655)
Supplement: Supplementary file 1 [file DataSheet1.pdf]

mRNA-engineered T lymphocytes secreting bispecific T cell engagers with therapeutic potential in solid tumors

SUPPLEMENTAL MATERIAL

Supplementary figures

Supplementary figure 1

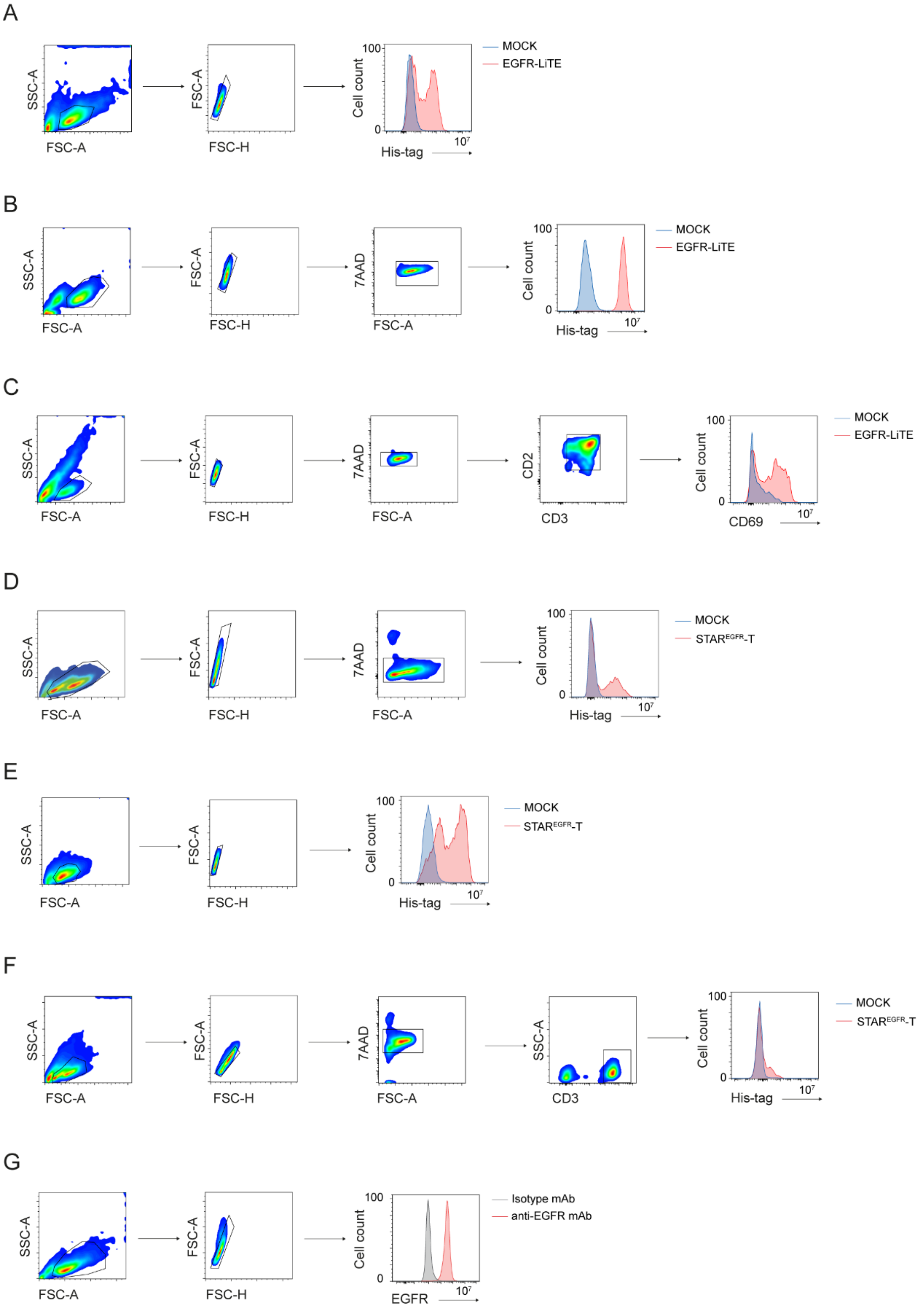

Supplementary figure 1. Flow cytometry gating strategies. (A) Transfection efficiency of EGFR-LiTE mRNA in 293T cells assessed by intracellular His-tag staining. (B) Binding of secreted EGFR-LiTE to $CD3^{+}$, $EGFR^{+}$, or double-negative cell lines. (C) CD69 expression in $CD3^{+}CD2^{+}$ Jurkat cells in activation assays. (D, E) Transfection efficiency of EGFR-LiTE mRNA–electroporated primary T cells assessed by surface (D) or (E) intracellular His-tag staining. (F) CD69 expression in $CD3^{+}$ primary T cells in activation assays. (G) EGFR expression in tumor cell lines.

Supplementary figure 2

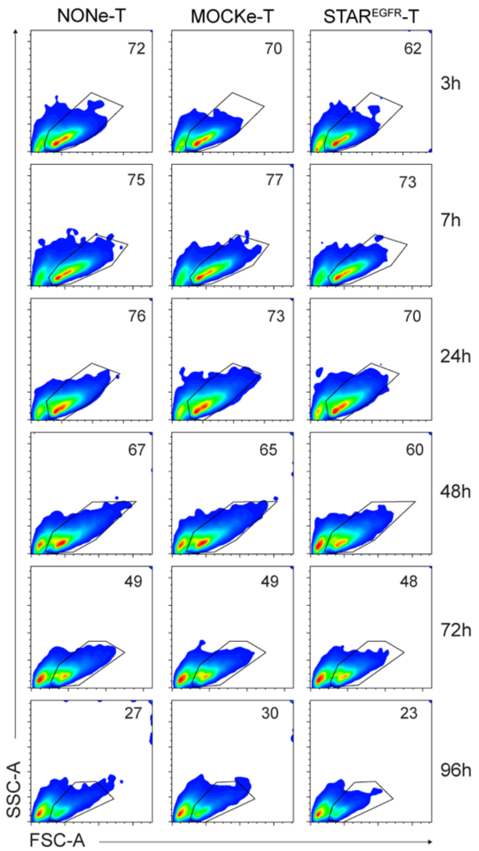

Supplementary Figure 2. Forward scatter vs. side scatter plots. Raw data showing all events in non-electroporated (NONe), MOCK electroporated (MOCKe) and EGFR-LiTE electroporated ($STAR^{EGFR}$-T) T cells at different time points post-electroporation. One representative experiment of three independent experiments is shown.

Supplementary figure 3

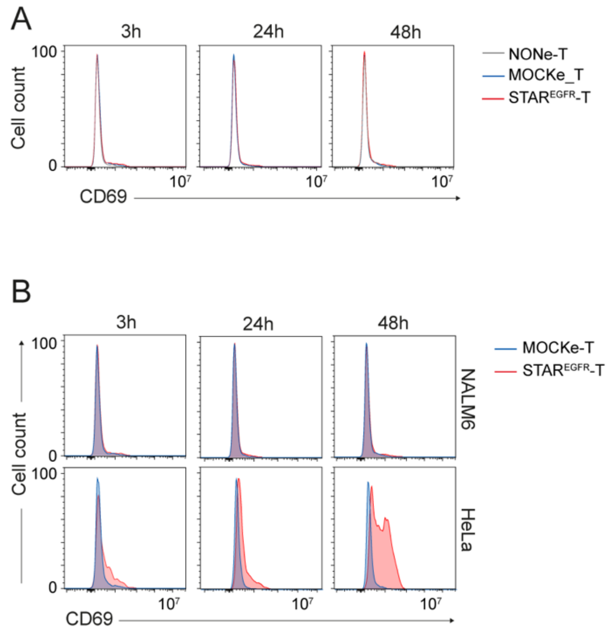

Supplementary Figure 3. Expression of CD69 marker upon electroporation. (A) Expression of CD69 on NONe-, MOCKe or STAR^{EGFR}-T cells cultured alone, at different time points upon electroporation. (B) Expression of CD69 on MOCKe- or STAR^{EGFR}-T cells collected at different times after electroporation and co-cultured for 24 hours with HeLA (EGFR^{+}) or NALM6 (EGFR^{-}) target cells. One representative experiment out of six independent experiments is shown.

Supplementary figure 4

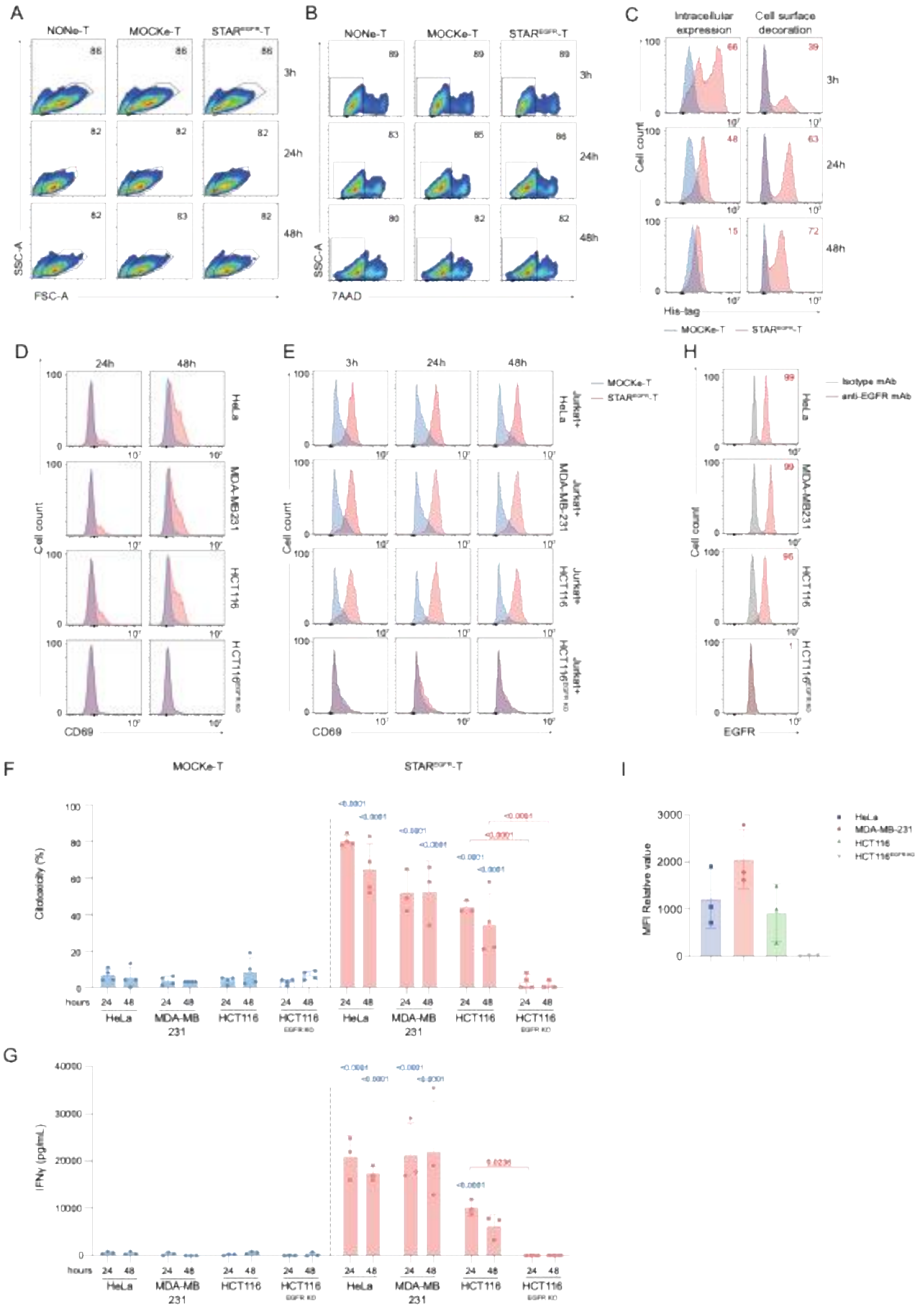

Supplementary Figure 4. STAR^{EGFR}-T cells kill different EGFR-expressing tumor cell lines. Human primary T cells were expanded for 6 days and either left non-electroporated (NONe-T), MOCK electroporated (MOCKe-T) or electroporated with EGFR-LiTE encoding mRNA (STAR^{EGFR}-T). Cells and cell culture supernatants were collected at 3, 24 and 48 hours post-electroporation. **(**A) Raw data showing all events in flow cytometry acquisition. (B) Percentage of viable (7AAD^{-}) NONe-, MOCKe- and STAR^{EGFR}- T cells. (C) Intracellular expression and cell-surface bound EGFR-LiTE (decoration) in STAR^{EGFR}-T cells (pink line) at the different time points after electroporation. MOCKe-T cells (blue line) were used as negative controls. (D) MOCKe- or STAR^{EGFR}-T cells were co-cultured with HeLa, MDA-MB-231 or HCT116 EGFR^{+} cells, or with HCT-116$^{EGFR\ KO}$ (EGFR^{-}) cells at an effector:target ratio (E:T) 2:1. After 24 hours, CD69 expression on T cells was analyzed by flow cytometry. (E) Unmodified Jurkat T cells were co-cultured for 24 hours with the different target cells at 2:1 E:T ratio in the presence of supernatants from MOCKe-T or STAR^{EGFR}-T cells collected at different time points. Expression of CD69 activation was analyzed by flow cytometry. (A-E) One representative experiment of four independent experiments is shown. (F,G) MOCKe- or STAR^{EGFR}-T cells (1x10^{5}) collected at different post-electroporation times were co-cultured with 5x10^{4} EGFR^{-} or EGFR^{+} luciferase-expressing cells. After 48 hours, (F) the percentage of specific cytotoxicity and (G) the level of IFNγ secretion were determined. Data represent mean ± SD of four independent experiments performed in triplicate. Significance was calculated by a two-way ANOVA test corrected with a Tukey´s multiple comparisons test. Descriptive statistics (mean,

SD, CV) are provided in Supplementary Tables 5 and 6. ANOVA, analysis of variance; MOCKe-T, mock-electroporated; $STAR^{EGFR}$-T, EGFR-LiTE electroporated. (H,I) Analysis of EGFR expression by the different cell lines used as target cells. (H) One representative experiment is shown. (I) Data represent mean ± SD of three independent experiments.

Supplementary figure 5

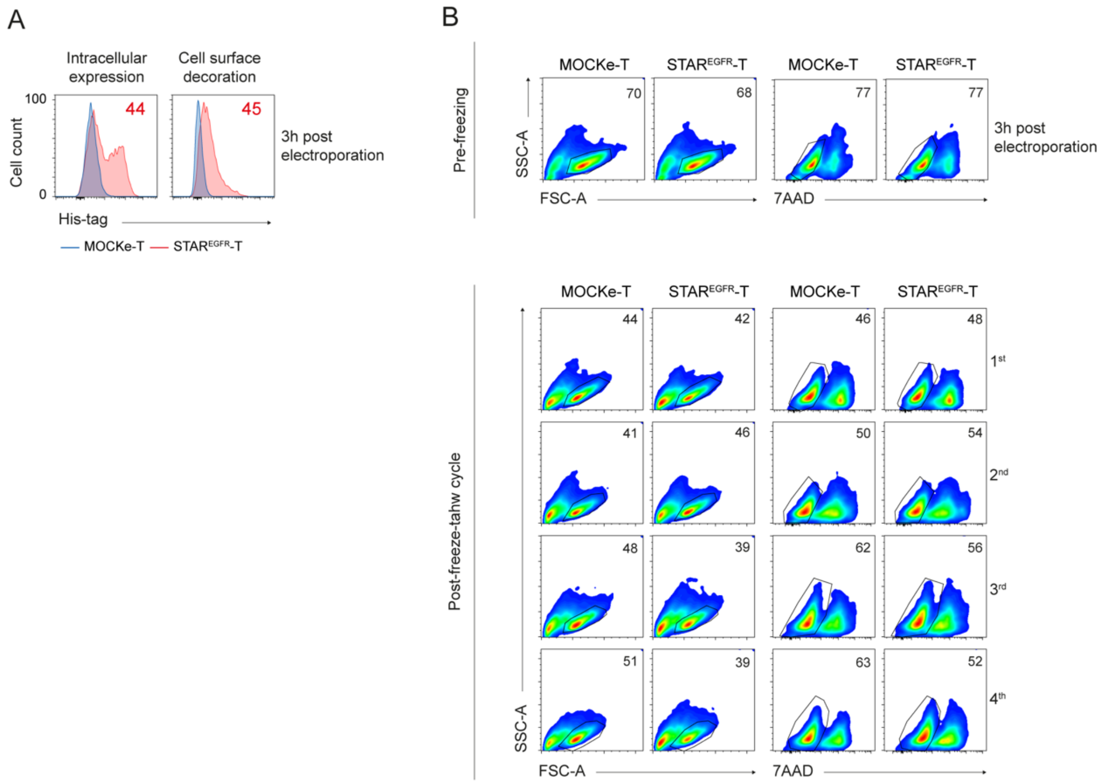

Supplementary Figure 5. EGFR-LiTE expression and viability of STAR-T cells engineered for high-dose HeLa xenograft model. (A) Intracellular expression and cell-surface bound EGFR-LiTE in STAR^{EGFR}-T cells (pink line) 3h after electroporation, immediately before freezing. MOCKe-T cells (blue line) were used as negative controls (n=1). (B) Raw data showing all events and percentage of viable (7AAD^{-}) non-electroporated (NONe), MOCK electroporated (MOCKe) and EGFR-LiTE electroporated (STAR^{EGFR}-T) T cells, 3 hours post-electroporation and 24 hours after thawing of the different doses injected (n=1).

Supplementary figure 6

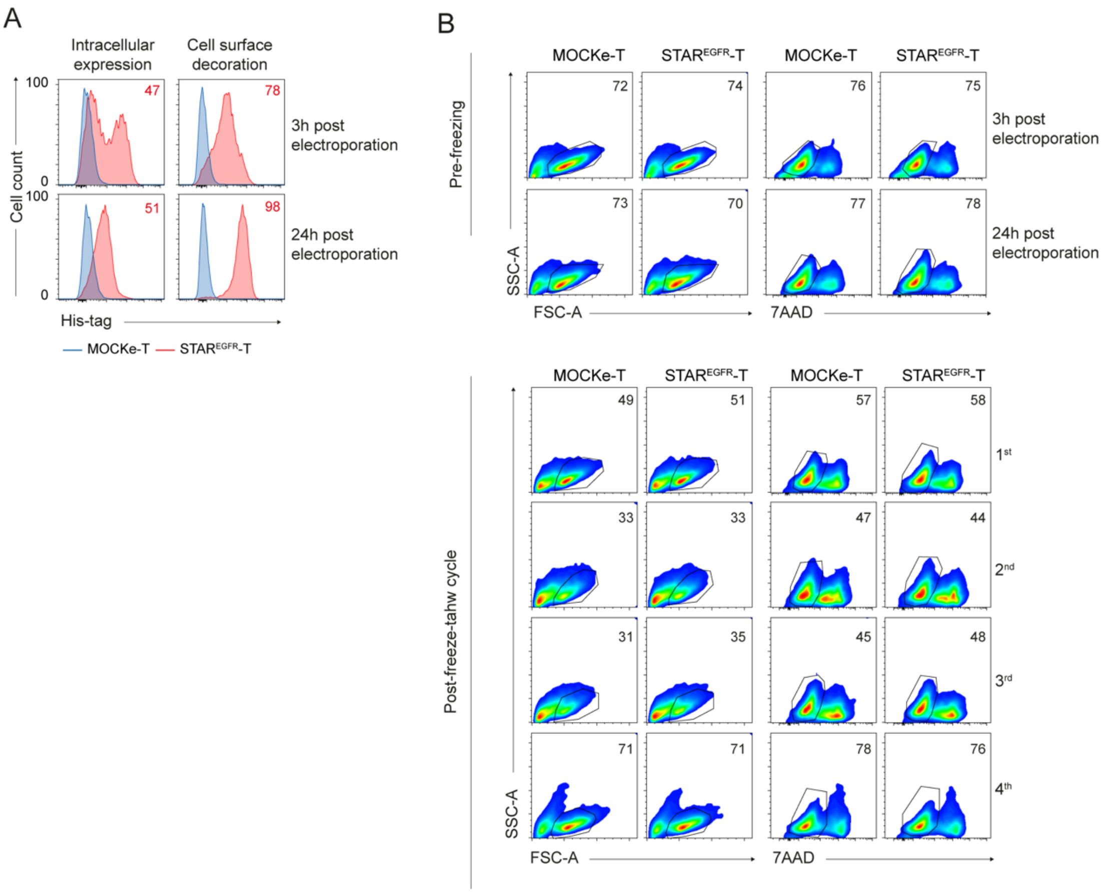

Supplementary Figure 6. **EGFR-LiTE expression and viability of STAR-T cells engineered for low-dose HeLa xenograft model.** (A) Intracellular expression and cell-surface bound EGFR-LiTE in STAR^{EGFR}-T cells (pink line) cells at 3 hours and 24 hours upon electroporation. MOCKe-T cells (blue line) were used as negative controls (n=1). (B) Raw data showing all events and percentage of viable (7AAD^{-}) non-electroporated (NONe), MOCK electroporated (MOCKe) and EGFR-LiTE electroporated (STAR^{EGFR}-T) T cells, 3 hours and 24 hours upon electroporation (pre-freezing) and 24 hours after thawing of the different doses injected (post freeze-thaw cycle) (n=1).

Supplementary figure 7

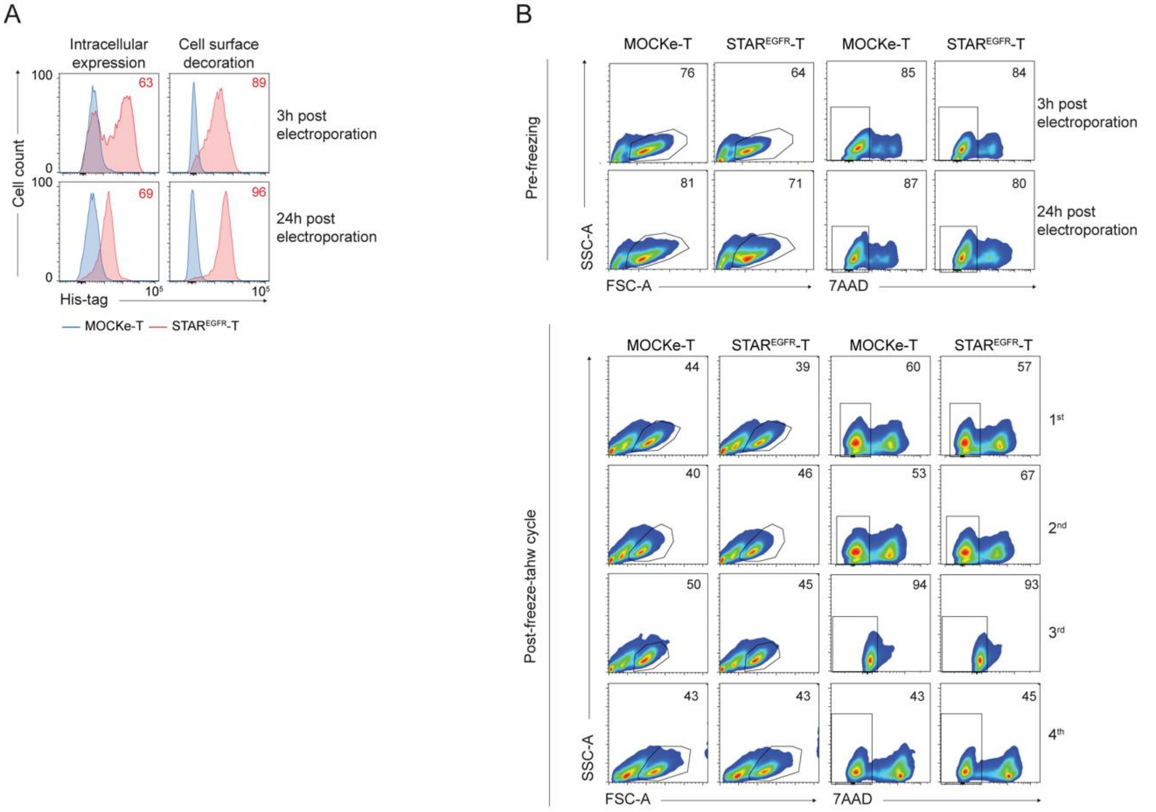

Supplementary Figure 7. **EGFR-LiTE expression and viability of STAR-T cells engineered for MDA-MB-231 xenograft model.** (A) Intracellular expression and cell-surface bound EGFR-LiTE in STAR^{EGFR}-T cells (pink line) cells at 3 hours and 24 hours upon electroporation. MOCKe-T cells (blue line) were used as negative controls (n=1). (B) Raw data showing all events and percentage of viable (7AAD^{-}) non-electroporated (NONe), MOCK electroporated (MOCKe) and EGFR-LiTE electroporated (STAR^{EGFR}-T) T cells, 3 hours and 24 hours upon electroporation (pre-freezing) and 24 hours after thawing of the different doses injected (post freeze-thaw cycle) (n=1).

Supplementary tables

Table 1: Analysis of variability of transfection efficacy (Figure 2D)

	Time	n	Mean	SD	CV (%)
Cell surface decoration	3h	6	43.93	26.53	60.39
	7h	6	85.03	11.81	13.88
	24h	6	90.38	5.534	6.123
	48h	6	87.25	9.88	11.32
	72h	2	71.9	21.35	29.70
	96h	1	80	0	0.000
Intracellular expression	3h	6	48	10.41	21.69
	7h	6	64.43	15.62	24.25
	24h	6	62.25	20.8	33.42
	48h	6	21.73	13.78	63.41
	72h	2	19.18	15.31	79.84
	96h	1	25	0	0.00

Table 2: Analysis of variability of EGFR-LiTE secretion (Figure 2E)

Time	Condition	n	Mean	SD	CV (%)
7h	MOCKe	3	0.07	0.009	12.45
	$STAR^{EGFR}$-T	3	0.79	0.461	58.41
24h	MOCKe	3	0.052	0.013	24.64
	$STAR^{EGFR}$-T	3	1.659	0.595	35.85
48h	MOCKe	3	0.058	0.017	28.49
	$STAR^{EGFR}$-T	3	0.516	0.067	13.01
72h	MOCKe	3	0.049	0.015	29.94
	$STAR^{EGFR}$-T	3	0.269	0.039	14.60
96h	MOCKe	3	0.046	0.019	41.30
	$STAR^{EGFR}$-T	3	0.243	0.085	34.85

Table 3: Analysis of variability of cytotoxicity assays (Figure 3A)

Time	Condition		n	Mean	SD	CV (%)
3 h	MOCKe	NALM6	6	6.83	3.43	50.2
		HeLa	6	6.50	2.35	36.1
	STAR^{EGFR}-T	NALM6	6	10.17	13.63	134.1
		HeLa	6	79.67	16.1	20.2
24 h	MOCKe	NALM6	6	6.50	3.89	59.8
		HeLa	6	4.50	2.35	52.1
	STAR^{EGFR}-T	NALM6	6	17.5	20.95	119.7
		HeLa	6	91.17	6.77	7.42
48 h	MOCKe	NALM6	6	5.67	2.66	46.9
		HeLa	6	3.33	2.34	70.1
	STAR^{EGFR}-T	NALM6	6	13.17	10.42	79.1
		HeLa	6	86.0	11.66	13.6
72 h	MOCKe	NALM6	1	7.0	0.0	0.0
		HeLa	1	2.0	0.0	0.0
	STAR^{EGFR}-T	NALM6	1	0.0	0.0	0.0
		HeLa	1	98.0	0.0	0.0

Table 4: Analysis of variability of IFN-γ secretion (Figure 3B)

Time	Condition		n	Mean	SD	CV (%)
3 h	MOCKe	NALM6	3	2045	1505	73.62
		HeLa	3	1988	2272	114.3
	$STAR^{EGFR}$-T	NALM6	3	2448	2184	89.21
		HeLa	3	222244	65956	29.68
24 h	MOCKe	NALM6	3	563.2	490.5	87.09
		HeLa	3	1253	1086	86.68
	$STAR^{EGFR}$-T	NALM6	3	963.1	989.4	102.7
		HeLa	3	133653	26680	19.96
48 h	MOCKe	NALM6	3	712.9	552.7	77.53
		HeLa	3	1506	1886	125.3
	$STAR^{EGFR}$-T	NALM6	3	693.3	793	114.4
		HeLa	3	52001	54367	104.5
72 h	MOCKe	NALM6	2	1333	936.7	70,29
		HeLa	2	1063	1503	141.4
	$STAR^{EGFR}$-T	NALM6	2	793.6	108.1	13.62
		HeLa	2	114353	150539	131.6

Table 5: Analysis of variability of cytotoxicity assays (Figure S4F)

Time	Condition		n	Mean	SD	CV (%)
24 h	MOCKe	HeLa	4	6.75	3.403	50.42
		MDA-MB-231	4	3.5	2.38	68.01
		HCT116	4	3.75	1.893	50.48
		HCT116 KO	4	3	1.414	47.14
	STAR^{EGFR}-T	HeLa	4	80.5	3.109	3.862
		MDA-MB-231	3	52	11.79	22.67
		HCT116	3	44	3.464	7.873
		HCT116 KO	4	3	3.83	127.7
48 h	MOCKe	HeLa	4	5.25	5.5	104.8
		MDA-MB-231	4	3	0	0
		HCT116	4	8.5	7.853	92.39
		HCT116 KO	4	6.5	2.38	36.62
	STAR^{EGFR}-T	HeLa	4	64.75	14.24	22.00
		MDA-MB-231	3	52.67	16.65	31.62
		HCT116	4	34.25	17.25	50.37
		HCT116 KO	3	1.333	2.309	173.2

Table 6: Analysis of variability of IFN-γ secretion (Figure S4G)

Time	Condition		n	Mean	SD	CV (%)
24 h	MOCKe	HeLa	3	626,3	231,4	36,96
		MDA-MB-231	3	383,8	365,3	95,17
		HCT116	3	181,8	157,5	86,60
		HCT116 KO	3	40,4	69,98	173,2
	$STAR^{EGFR}$-T	HeLa	3	20929	4480	21,41
		MDA-MB-231	3	21232	6790	31,98
		HCT116	3	10172	1620	15,93
		HCT116 KO	3	40,4	69,98	173,2
48 h	MOCKe	HeLa	3	525,3	315,4	60,05
		MDA-MB-231	3	0	0	0,00
		HCT116	3	676,8	231,4	34,20
		HCT116 KO	3	282,8	389,6	137,8
	$STAR^{EGFR}$-T	HeLa	3	17343	1555	8,966
		MDA-MB-231	3	21939	10821	49,32
		HCT116	3	6232	2541	40,78
		HCT116 KO	3	121,2	0	0,00
